# Identification of DNMT3B2 as the Predominant Isoform of DNMT3B in Porcine Alveolar Macrophages and Its Involvement in LPS-Stimulated TNF-α Expression

**DOI:** 10.3390/genes11091065

**Published:** 2020-09-10

**Authors:** Yanbing Zhang, Hui Li, Xiao Xiang, Yan Lu, Mona Sharma, Zongjie Li, Ke Liu, Jianchao Wei, Donghua Shao, Beibei Li, Zhiyong Ma, Yafeng Qiu

**Affiliations:** Shanghai Veterinary Research Institute, Chinese Academy of Agricultural Sciences, Shanghai 200241, China; zhangyanbing129@outlook.com (Y.Z.); lihui022715@outlook.com (H.L.); xiao.xiang@wur.nl (X.X.); yanlu013194@outlook.com (Y.L.); monasharma1990@yahoo.com (M.S.); lizongjie@shvri.ac.cn (Z.L.); liuke@shvri.ac.cn (K.L.); jianchaowei@shvri.ac.cn (J.W.); shaodonghua@shvri.ac.cn (D.S.); lbb@shvri.ac.cn (B.L.); zhiyongma@shvri.ac.cn (Z.M.)

**Keywords:** porcine alveolar macrophages, DNMT3B, DNA methylation, isoform, TNF-α

## Abstract

DNA methyltransferase 3B (DNMT3B) as one member of the DNMT family functions as a de novo methyltransferase, characterized as more than 30 splice variants in humans and mice. However, the expression patterns of DNMT3B in pig as well as the biological function of porcine DNMT3B remain to be determined. In this study, we first examined the expression patterns of DNMT3B in porcine alveolar macrophages (PAM). We demonstrated that only DNMT3B2 and DNMT3B3 were the detectable isoforms in PAM. Furthermore, we revealed that DNTM3B2 was the predominant isoform in PAM. Next, in the model of LPS (lipopolysaccharide)-activated PAM, we showed that in comparison to the unstimulated PAM, (1) expression of DNTM3B is reduced; (2) the methylation level of TNF-α gene promoter is decreased. We further establish that DNMT3B2-mediated methylation of TNF-α gene promoter restricts induction of TNF-α in the LPS-stimulated PAM. In summary, these findings reveal that DNMT3B2 is the predominant isoform in PAM and its downregulation contributes to expression of TNF-α via hypomethylation of TNF-α gene promoter in the LPS-stimulated PAM.

## 1. Introduction

DNA methyltransferase 3B (DNMT3B) is one member of the DNMT family which comprises DNMT1, DNMT3A, DNMT3B, as well as DNMT3L (DNMT3-like) in mammals. In mammalian systems, DNMT3B similar with DNMT3A serves as de novo methyltransferase for the establishment of DNA methylation [1]; in comparison, DNMT1 acts as maintenance of DNA methylation [2]. Moreover, DNMT3L has no DNA methyltransferase activity, but it can act as an accessory factor of the other DNMTs to regulate DNA methylation [3]. Although the functional characteristics between DNMT3A and DNMT3B are similar, the expression patterns of them are very different, i.e., in comparison to two isoforms of mouse DNMT3A [4], more than 30 isoforms of DNMT3B are identified in humans and mice [5,6].

Although there are more than 30 isoforms of DNMT3B, the expression patterns of DNMT3B appear to be highly conserved, at least in humans and mice. For example, human DNMT3B2 has a 60 bp-deletion (representative of exon 10) in comparison to the canonical isoform DNMT3B1 [5,7]; furthermore, DNMT3B3 of humans and mice has two deletions including a 60-bp deletion and a 189-bp deletion (representative of exon 21 and 22) in comparison to DNMT3B1 [5,8]. Notable, the alternative splicing of DNMT3B could influence DNMT3B functions. Thus, to clarify the expression patterns of DNMT3B is important to understand DNMT3B functions in the other species like pig.

Given the reported data, many spliced variants of DNMT3B are expressed in a tissue, cell, and/or developmental stage-specific manner [6,9], in this study, we focus on the expression pattern of DNMT3B in porcine alveolar macrophages (PAM), which are not only the major immune cells in pig lungs, but also the important resources of the inflammatory cytokines in pneumonia of pigs [10,11]. Though it has been shown that DNA methylation plays role in modulation of lipopolysaccharide (LPS)-induced inflammation in PAM [12], little is known about how DNMT3B is involved in this process. Furthermore, we determined effects of DNA methylation regulated by DNMT3B on TNF-α expression in the LPS-activated PAM. In this study, for the first time we determine the expression pattern of DNMT3B and clarify its effect on TNF-α expression in the LPS-stimulated PAM.

## 2. Materials and Methods

### 2.1. Piglets and Porcine Alveolar Macrophages (PAM)

Clinical healthy 35-day-old piglets (Shanghai great white pig strain) were purchased from the Shanghai Academy of Agricultural Sciences (Shanghai, China). All animal experiments were approved by the Institutional Animal Care and Use Committee of Shanghai Veterinary Research Institute (IACUC No: Shvri-po-201606 0501) and were performed in compliance with the Guidelines on the Humane Treatment of Laboratory Animals (Ministry of Science and Technology of the People’s Republic of China, Policy No.2006 398). Porcine alveolar macrophages (PAM) were isolated from piglets as previously described [13] and cultured in RPMI 1640 containing 10% FBS, penicillin, streptomycin, GlutaMAX (all purchased from Thermo Fisher Scientific, Shanghai, China).

### 2.2. Cloning of Porcine DNMT3B Isoforms and Sequence Analysis

Total RNA was extracted from PAM (at least 1.0 × 10^6^ cells) by using Trizol method [14] (Takara Biotechnology, Dalian, China), and cDNA was prepared by using Super ScriptII Reverse Transcriptase (Thermo Fisher Scientific, Shanghai, China). Three pairs of primers (Table S1) used to amplify full-length porcine DNTM3B were designed based on the porcine DNMT3B1 sequence deposited in GenBank (XM_013985274.2). All PCR products were cloned using pMD19-T Vector Cloning Kit (Takara Biotechnology, Dalian, China), then positive clones were selected and sequenced by using M13 (Bacteriophage M13) forward and reverse primers. The sequences of DNMT3B2 and DNMT3B3 were obtained and deposited in NCBI GenBank with accession numbers MN873575 and MN207312, respectively. Subsequently, porcine DNMT3B2 or DNMT3B3 was inserted into the p3 × Flag-CMV-14 vector (Sigma, St. Louis, MO, USA) by homologous recombination with the ClonExpress MultiS One Step Cloning Kit (Vazyme Biotech, Nanjing, China) (Table S1), named Flag-DNMT3B2 or DNMT3B3, respectively.

Furthermore, the expression of exon 10 was analyzed by RT-PCR (reverse transcription-PCR, RT-PCR) with an exon 9 forward primer and an exon 11 reverse primer (Table S1). Moreover, PCR was performed to detect the presence of exon 10 in genomic DNA of PAM with an intron 9 forward primer and an intron 10 reverse primer (Table S1). Furthermore, semi-quantitative RT-PCR was performed with an exon 20 forward primer and an exon 23 reverse prime (Table S1) to analyze the expression profile of DNMT3B2 and DNMT3B3 in PAM. All PCR products were sequenced using gene-specific primers. All of the images for agarose gel electrophoresis were captured by image lab version 5.1 (Bio-Rad Laboratories, Hercules, CA, USA).

The amino acid sequence alignment of human (NP_008823.1), mouse (NP_001003961.2), and three porcine DNMT3B isoforms was performed using the Clustal V method and edited using Genedoc. The phylogenetic tree was constructed using the available DNMT3B proteins by the neighbor-joining method with 1000 bootstrap replicates in MEGA version 6.06 [15,16]

### 2.3. Generation of Polyclonal Antibodies against Porcine DNMT3B

Rabbit experiments were approved by the Institutional Animal Care and Use Committee of Shanghai Veterinary Research Institute (IACUC No: Shvri-po-201606 0501) and were performed in compliance with the Guidelines on the Humane Treatment of Laboratory Animals (Ministry of Science and Technology of the People’s Republic of China, Policy No.2006 398). Rabbit against porcine DNMT3B antibodies were generated according to a previous publication [17]. Briefly, a peptide of 15 amino acids (SYTQDLTGDGDGEGE) residues of the porcine DNMT3B was synthesized. Rabbits were immunized five times with the peptide in combination with complete or incomplete Freund’s adjuvants every 14 days.After 7 days of the fifth immunization, rabbits were euthanized and serum was generated. The specificity of the generated DNMT3B antibodies was tested by Western blot.

### 2.4. Western Blot

The protein samples were prepared from the cell pellets as previously described [18,19]. Then the samples were separated on SDS-PAGE gel and transferred to NC (nitrocellulose, NC) membrane. After blocking in 5% nonfat milk, primary antibodies were added and incubated with membrane overnight at 4 °C (anti-Flag (1:5000, M2, Sigma), anti-DNMT3B (1:1000), anti-actin (1:10,000, clone C4, Sigma)). Secondary antibody was incubated for 1 h at room temperature (goat anti-mouse HRP (1:5000, Abcam), goat anti-rabbit (1:10,000, Abcam)). The images of Western blot were captured by image lab version 5.1 (Bio-Rad Laboratories, Hercules, California, USA).

### 2.5. Quantitative Real-Time Reverse Transcription-PCR (qRT-PCR)

cDNA was prepared using PrimeScript RT Reagent Kit including gDNA Eraser (Takara, Dalian, China). Then RT-PCR was conducted using a SYBR Premix Ex Taq kit (Takara, Dalian, China). Specific primers are shown in Table S1. The calculation was performed as described in our previous study [20].

### 2.6. Bisulfite Sequencing PCR (BSP)

Genomic DNA was extracted from PAM treated with LPS (1 μg/mL) and vehicle for 6 h. Then genomic DNA (0.8 μg) was subjected to bisulfite treatment using EZ DNA Methylation-Gold Kit (ZymoResearch, LA, USA). Primers for Bisulfite sequencing PCR (BSP) (Table S1) were designed based on porcine *TNF*-*α* gene promoter sequence using online software (http://www.urogene.org/cgi-bin/methprimer/methprimer.cgi) [21]. Subsequently, BSP [22] was performed using EpiMark Hot Start Taq DNA Polymerase (New England Biolabs, Ipswich, MA, USA) following the manufacturer’s protocol. Briefly, using BSP primers amplified the region of TNF-α promoter, running an agarose gel to recover the PCR products. PCR products were cloned into the pMD19-T vector (Takara, Dalian, China). More than 10 positive clones were randomly selected for DNA sequencing [23,24]. The sequencing data and non-CpG-C-T conversion rates were analyzed using online QUMA software (http://quma.cdb.riken.jp/top/index.html) [25]. The total percentage of methylated CpG was calculated in each group including vehicle-treated, LPS-treated, vector-transfected, and DNMT3B2-transfected groups. Additionally, the difference of methylation level between certain groups was analyzed using Fisher’s exact test of the online QUMA software.

### 2.7. Lentivirus Production

HEK293T cells were cultured in Dulbecco’s modified Eagle’s medium (DMEM) containing 10% FBS, penicillin, streptomycin (Thermo Fisher Scientific, Shanghai, China). The pLenO-DCE-DNMT3B2 or pLenO-DCE-Vector (Invabio, Shanghai, China) was co-transfected with pRsv-REV, pMDlg-pRRE, pMD2G (Addgene) into HEK293T cells using Lipofectamine 2000 reagent (Invitrogen, Carlsbad, CA, USA). The supernatants were collected at 72 h post-transfection and concentrated through ultra-centrifugation (25,000 rpm, 4 °C, 2 h, L7 Ultracentrifuge, Beckman, Duarete, CA, USA) after filtering through a 0.45 μm syringe filter [26,27].

### 2.8. Statistical Analysis

All data shown are arithmetic means ± standard deviations. Statistical significance was assessed using unpaired Student’s *t*-test by GraphPad Prism software version 5.01 (GraphPad Software, Inc., La Jolla, CA, USA). ‘n’ refers to the sample size.

## 3. Results

### 3.1. Identification of DNMT3B2 and DNMT3B3 as the Detectable Isoforms in Porcine Alveolar Macrophages (PAM)

**Figure 1:**
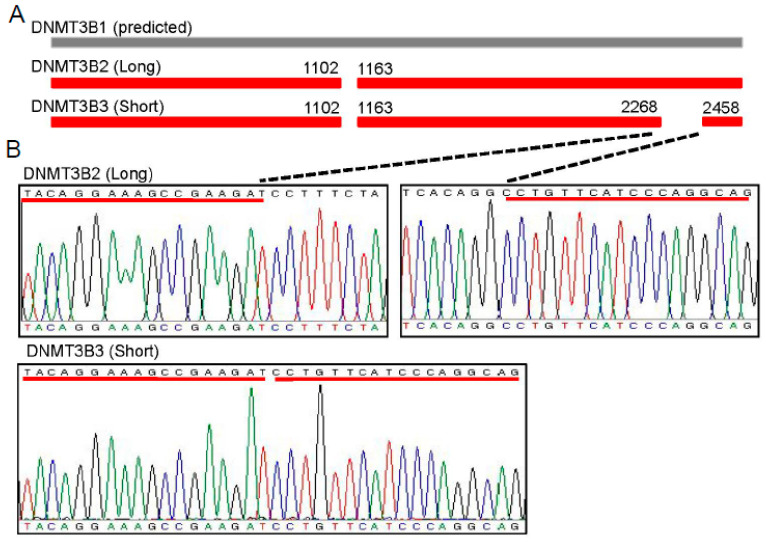

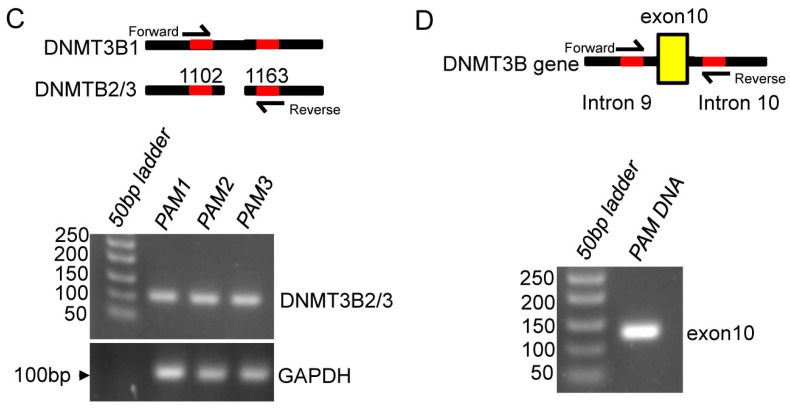
Identification of two splice variants of DNA methyltransferase 3B (DNMT3B) in porcine alveolar macrophages (PAM). (**A**) Schematic representation of the predicted DNMT3B1 and two splice variants identified in PAM. (**B**) Sequencing chromatogram showing the deleted region in the short splice variant named DNMT3B3 in comparison with the long splice variant named DNMT3B2. (**C**) Expression analysis of exon 10 of DNTM3B and GAPDH (about 90 bp) in PAM cDNA. Schematic representation of the exon 10-including fragment (about 160 bp) and the exon 10-excluded fragment (about 100 bp) from DNMT3B1 and DNMT3B2/3, respectively. Note that only a short fragment was obtained, indicating that expression of exon 10 of DNMT3B was absent in PAM. (**D**) As shown in the schematic, PCR was performed with an intron 9-forward primer and an intron 10-reverse primer to identify the presence of exon 10 (the positive fragment with 121 bp) in the genomic DNA from PAM. Note that a unique fragment was obtained, which was confirmed by sequencing analysis to show the presence of exon 10 in the *DNMT3B* gene of PAM.

According to the predicted porcine *DNMT3B1* gene sequence (GenBank accession number: XM_013985274.2), we designed the primers to amplify the DNMT3B ORF in PAM cDNA. Interestingly, the full-length sequencing results showed that only DNMT3B2 (GenBank accession number, MN873575) and DNMT3B3 (GenBank accession, MN207312) were identified in PAM (Figure 1A,B). Given that alternative splicing of DNMT3B exon 10 [5,7] distinguished DNMT3B1 (exon 10-included isoforms) with DNMT3B2 and DNMT3B3 (the exon 10-excluded isoforms), we further investigated the expression of DNMT3B exon 10 in PAM. In comparison to the expected fragment (about 160 bp) containing exon 10, a short fragment (about 100 bp) was obtained (Figure 1C). Moreover, the sequencing analysis confirmed that DNMT3B exon 10 was absent in the PCR product. We also investigated the presence of exon 10 in *DNMT3B* gene in PAM by PCR. Successfully, we obtained the fragment containing exon 10 (Figure 1D) which was further confirmed by the sequencing analysis. Taken together, these data reveal that expression of DNMT3B exon 10 is lost in PAM. Consistently, DNMT3B2 and DNMT3B3, the exon 10-excluded isoforms, are detectable in PAM.

### 3.2. Identification of DNMT3B2 as the Predominant Isoform in Porcine Alveolar Macrophages

**Figure 2:**
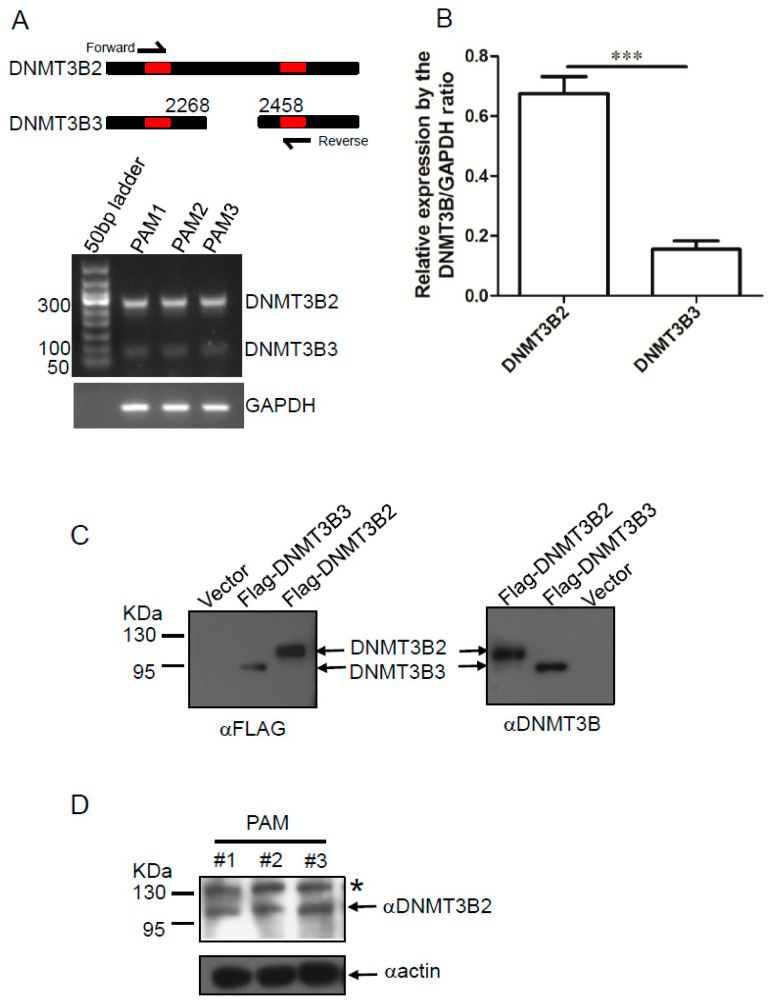
(**A**) Total RNA was extracted from PAM and analyzed by RT-PCR with exon 20 forward primer and an exon 23 reverse primer for the expression profile of DNMT3B2 and DNMT3B3 in PAM. Note that two fragments were obtained: one is more than 250 bp, representative expression of DNMT3B2; another is less than 100 bp, representative of DNMT3B3. (**B**) Furthermore, the bar graph represents relative expression of DNMT3B2 or DNMT3B3 by calculating the DNMT3B2/GAPDH ratio. The data shown are mean (SD) (*n* = 3), ***, *p* < 0.001. (**C**) HEK 293T cells were transiently transfected with recombinant plasmids expressing Flag-pDNMT3B2 and Flag-pDNMT3B3, respectively. Cell lysates were analyzed by Western blot with anti-*p*DNMT3B or anti-Flag antibodies. (**D**) The expression profile of DNMT3B in PAM was analyzed by Western blot. Note that only DNMT3B2 was detected in PAM. * is representative of nonspecific reaction.

In comparison to DNMT3B2, we observed a 189-bp deletion in DNTM3B3 (Figure 1A,B), which is attributed to lack expression of exon 21 and exon 22 according to the previous reports [5,8]. Based on this expression pattern between DNMT3B2 and DNMT3B3, we set up the RT-PCR with an exon 20 forward primer and an exon 23 reverse prime to analyze the expression profile of DNMT3B2 and DNMT3B3 in PAM. As expected, we totally obtained two fragments by analysis of the RNA samples extracted from PAM: the long fragment (268 bp) is representative of expression level of DNMT3B2; the short fragment (79 bp) is representative of expression level of DNMT3B3 (Figure 2A). Notable, the expression abundance of DNMT3B2 looked greater than that of DNMT3B3 according to the results by agarose gel electrophoresis. The density calculation further confirmed that expression of DNMT3B2 was much higher than that of DNMT3B3 in PAM (Figure 2B). Furthermore, in order to determine the protein level of DNMT3B2 and DNMT3B3, we used an antigen peptide of DNMT3B to generate polyclonal antibodies against DNMT3B. Western blot showed that the polyclonal antibodies were able to specifically detect expression of porcine DNMT3B2 and DNMT3B3 (Figure 2C). Then, we used the polyclonal antibodies to detect the expression profile of DNMT3B in PAM. Our result showed that only DNMT3B2 was detected in PAM (Figure 2D). Collectively, our data reveal that DNMT3B2 is the predominant isoform in PAM.

### 3.3. Sequence Analysis of Porcine DNMT3B2 and DNMT3B3

**Figure 3:**
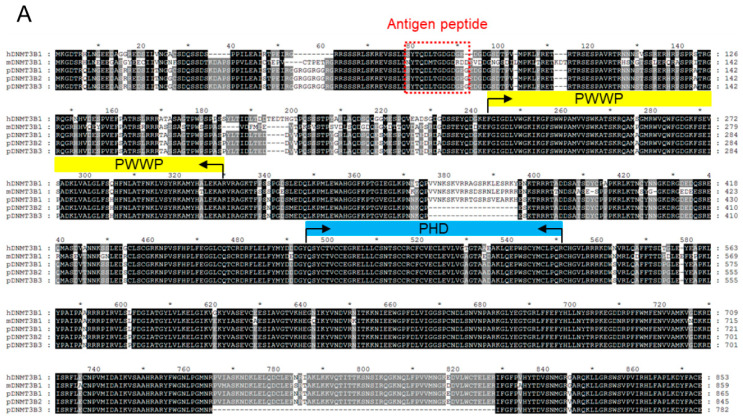

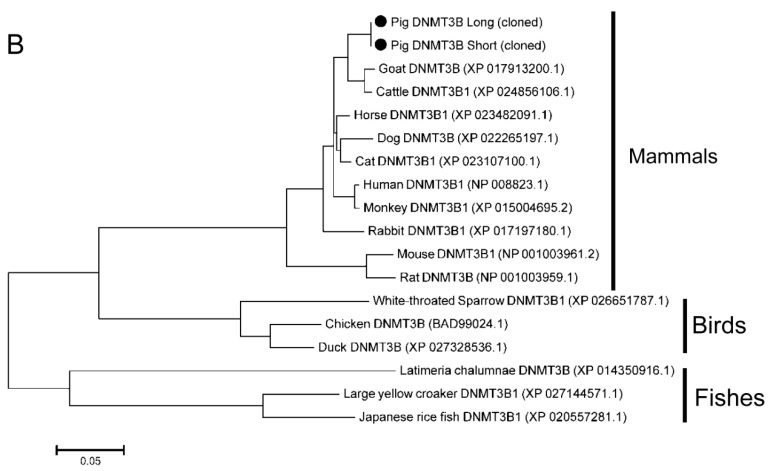
(**A**) Comparative sequence analysis of DNMT3B protein from different hosts. The black and gray shading highlight identity in all of the selected sequences and similarity, respectively. The PWWP (yellow line) and PHD (blue line) domains are labeled in reference to the human DNMT3B1 (NP_008823.1), respectively. Moreover, the antigen peptide is shown in the red box. (**B**) Phylogeny analysis of DNMT3B protein from different hosts. The scale bar indicates the genetic distance.

Having demonstrated that porcine DNMT3B2 and DNMT3B3 share the same expression pattern with that in humans and mice, we next performed sequence analysis to determine the sequence similarity and evolutional relationship of porcine DNMT3B with the other species. Multiple sequence alignment illustrated that the amino acid sequence and function domain of DNMT3B were conserved among human, mouse, and pig (Figure 3A). Notable, both PWWP domain (named as the well-conserved residues, Pro-Trp-Trp-Pro) and PHD domain (the plant homeodomain) in porcine DNMT3B are more than 94% identical to that of human and mouse (data not shown). Moreover, two deletion regions in the porcine DNMT3B3 were founded in comparison to DNMT3B1 of human, mouse and pig, which were attributed to alternative splicing of exon 10, exon 21, and exon 22. Furthermore, phylogenetical analysis of 18 protein sequences of DNMT3B classified them into three branches: mammals, birds, and fishes (Figure 3B), indicating that porcine DNMT3B is clustered with the other mammals.

### 3.4. Downregulation of DNMT3B Associates with the Demethylation of TNF-α Gene Promoter in the LPS-Activated PAM

**Figure 4:**
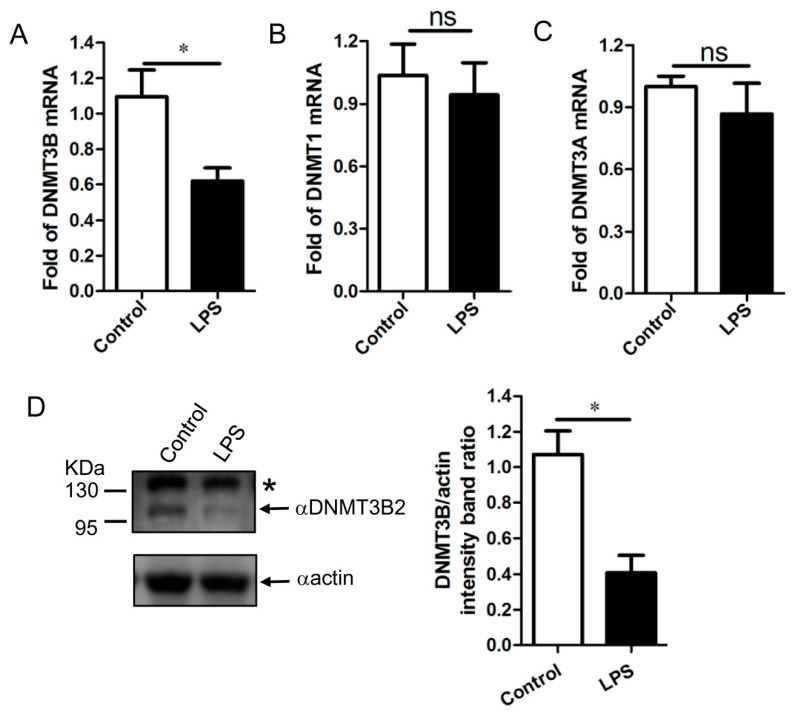
Total RNA was extracted from the treated and untreated PAM by LPS and expression of DNMT3B, DNMT1, and DNMT3A was analyzed by qRT-PCR analysis. The relative expression of DNMT3B (**A**), DNMT1 (**B**), and DNMT3A (**C**) was calculated as per material and method. *n* = 3, the data shown are mean (SD), ns is representative of no significant difference between control and LPS stimulated groups, *, *p* < 0.05. (**D**) PAM with or without LPS treatment was harvested and analyzed by Western blot with anti-pDNMT3B antibody. Note that LPS stimulation decreased expression of DNMT3B2 in comparison to control group. The representative bar graph was calculated by DNMT3B2/actin ration. The data shown are mean (SD) (*n* = 3), *, *p* < 0.05.

Furthermore, these results by sequence analysis motivated us to determine the biological function of porcine DNMT3B. Given the reported data that expression of DNMT3B is downregulated in LPS-activated mouse macrophages, which is associated with the global demethylation of DNA [28], thus we chose the LPS-activated PAM as our model for the functional analysis of porcine DNMT3B. Since the alteration of DNMT3B in the LPS-stimulated porcine alveolar macrophages remains unknown, we first investigated the expression profile of DNMT3B in the LPS-activated PAM. In comparison to the untreated PAM, the mRNA level and protein level of DNMT3B was significantly downregulated in LPS-stimulation PAM. Besides, the expression profile of DNMT1 and DNMT3A was also determined in the untreated and LPS-activated PAM (Figure 4A,D). Interestingly, expression of DNMT1 and DNMT3A were not affected by LPS stimulation compared with those in the unstimulated PAM (Figure 4B,C). Thus, these data demonstrated that expression of DNMT3B is reduced in LPS-activated PAM, which might involve in the demethylation of DNA during LPS stimulation.

**Figure 5:**
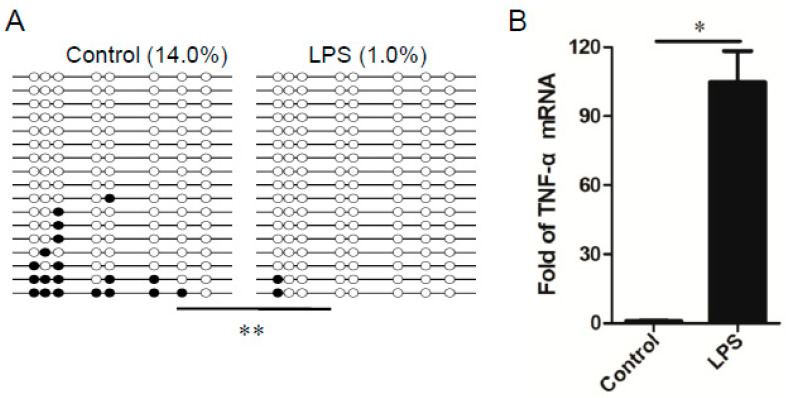
(**A**) The DNA methylation level of *TNF-α* promoter region was analyzed by Bisulfite sequencing PCR (BSP). Each line represents one individual sequenced clone. One circle represents one CpG site: the open circle shows unmethylated CpG; the black circle shows methylated CpG. Note that LPS stimulation significantly reduced the DNA methylation level of *TNF-α* promoter region in comparison to control groups. The difference between LPS group (*n* = 3) and control group (*n* = 3) was calculated by Fisher’s exact test, **, *p* < 0.01. (**B**) The relative expression of TNF-α in PAM with or without LPS treatment was analyzed by qRT-PCR. Note that LPS stimulation promoted TNF-α expression in comparison to control group. *n* = 3, the data shown are mean (SD), *, *p* < 0.05.

Given that the methylation status of *TNF-α* gene promoter plays a role in modulation of TNF-α expression [29,30,31], we next used the model of LPS-activated PAM to determine whether reduction of DNMT3B contributes to the demethylation of the *TNF-α* gene promoter. We performed Bisulfite sequencing PCR to examine the methylation profile of the *TNF-α* gene promoter region (−397 to −151) in the untreated and LPS-activated PAM, respectively. In comparison to the untreated PAM, the methylation level of *TNF-α* gene promoters in the LPS-activated PAM was significantly decreased (Figure 5A), which is accompanied with the robust induction of TNF-α in the stimulated PAM (Figure 5B). Thus, our data reveal that the demethylation of *TNF-α* gene promoter is associated with induction of TNF-α in the LPS-activated PAM.

### 3.5. DNMT3B2-Mediated Methylation of TNF-α Gene Promoter Restricts Induction of TNF-α in the LPS-Stimulated PAM

**Figure 6:**
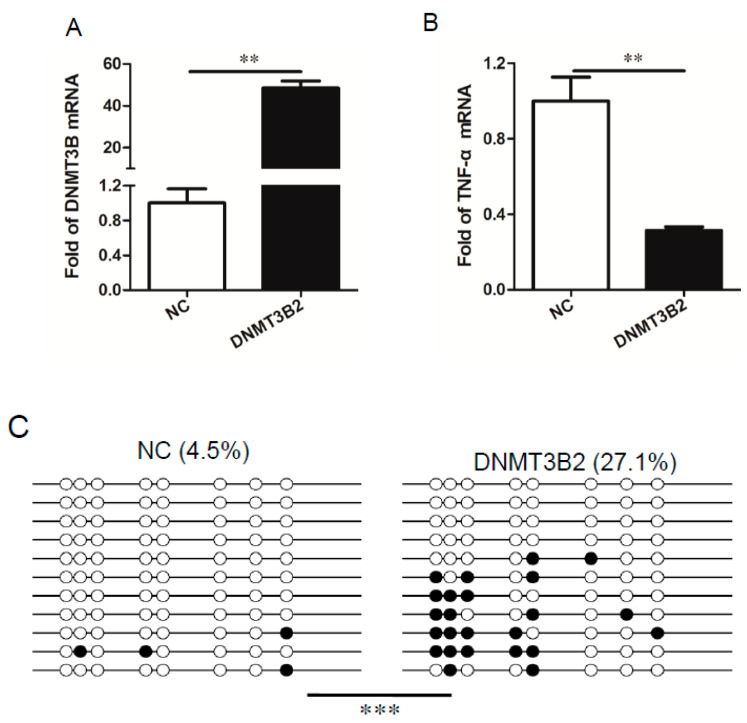
Effect of overexpression of DNMT3B2 by lentivirus on TNF-α transcription and methylation profile of *TNF-α* promoter. PAM were infected with lentivirus overexpressing DNMT3B2 or containing control vector. After 48 h of infection, cells were stimulated with LPS (1 μg/mL) for 6 h. Subsequently, cells were harvested, mRNA was extracted and analyzed for expression of DNMT3B2 (**A**) and TNF-α (**B**) by qRT-PCR, respectively. Data are shown as mean (SD) (*n* = 3). *, *p* < 0.05; **, *p* < 0.01. In the meanwhile, cells were treated as shown above, and the total DNA was collected and analyzed for the DNA methylation level of the *TNF-α* promoter region by BSP (**C**). Each line represents one individual sequenced clone. One circle represents one CpG site: the open circle shows unmethylated CpG; the black circle shows methylated CpG. Note that overexpression of DNMT3B2 significantly increased the DNA methylation level of the *TNF-α* promoter region in comparison to control group. The difference between DNMT3B2 group (*n* = 3) and control group (*n =* 3) was calculated by Fisher’s exact test, ***, *p* < 0.001.

Given that reduction of DNMT3B is associated with the demethylation of *TNF-α* gene promoter and that DNMT3B2 is the predominant isoform in PAM, therefore, our final goal was to determine the biological effect of DNMT3B2 on methylation of *TNF-α* gene promoter as well as expression of TNF-α in PAM. In order to do so, PAM were treated with lentivirus expressing porcine DNTMT3B2 and lentivirus vector (negative control, NC), respectively. After 24 h, the cells were stimulated with LPS and then harvested for the certain analysis. As shown in Figure 6A, in comparison to NC group, PAM treated with lentivirus expressing porcine DNMT3B2 showed a high level of DNMT3B even under the LPS-stimulated conditions. In contrast, the induced TNF-α was restricted by the increased DNMT3B2 in comparison to NC-treated PAM (Figure 6B). Furthermore, the methylation level of *TNF-α* gene promoter (−397 to −151) was analyzed in these two groups. Notable, PAM treated with lentivirus expressing porcine DNMT3B2 showed the higher methylation level of the *TNF-α* gene promoter than that in NC-treated PAM (Figure 6C). Thus, these results reveal that DNMT3B2-mediated methylation of *TNF-α* gene promoter modulates expression of TNF-α in the LPS-stimulated PAM.

## 4. Discussion

This study provides novel information regarding the expression pattern of DNMT3B in porcine alveolar macrophages and its effect on TNF-α expression in the LPS-activated PAM. Although there are more than 30 isoforms of DNMT3B identified in humans and mice, yet the expression pattern of DNMT3B in pigs remains to be determined. According to the predicted porcine DNMT3B1 gene sequence (GenBank accession number: XM_013985274.2), we sought to clone the different isoforms of DNMT3B in porcine alveolar macrophages (PAM). In this study, we demonstrate that DNMT3B2 and DNMT3B3 are the detectable isoforms in PAM. In contrast, the canonical isoform, DNMT3B1 and the other splice variants were not founded in the analysis. Since expression of DNMT3B exon 10 was not detected in PAM, alternative splicing of DNMT3B exon 10 [5,7] is important to result in the exon 10-included isoforms (DNMT3B1) and the exon 10-excluded isoforms (DNMT3B2 and DNMT3B3), respectively. Furthermore, we identified DNTM3B2 as the predominant isoform in PAM.

In the model of LPS-activated PAM, we show that in comparison to the unstimulated PAM, (1) expression of DNTM3B is reduced; (2) the methylation level of *TNF-α* gene promoter is decreased. We further establish that DNMT3B2-mediated methylation of *TNF-α* gene promoter restricts induction of TNF-α in the LPS-stimulated PAM. Collectively, our data identify DNMT3B2 as the predominant isoform to have a role in modulation of expression of TNF-α via DNA methylation in the LPS-stimulated PAM.

It has been reported that more than 30 DNMT3B isoforms are identified in humans and mice [5,6]. However, the expression patterns of porcine DNMT3B remains unknown. Here, we demonstrate that DNMT3B2 and DNMT3B3 are detectable in PAM; in comparison, DNMT3B2 is the predominant isoform. Furthermore, we demonstrate that the expression pattern of porcine DNMT3B2 and DNMT3B3 is same as what is shown in humans and mice [5,7,8], i.e., DNMT3B2 is lacking exon10; DNMT3B3 is lacking exon 10, exon 21, and exon 22. Thus, these results provide the evidence that the expression pattern of DNMT3B is conserved in different species [32].

Interestingly, we could not detect expression of DNMT3B1 in PAM despite the presence of exon 10 in the genome. In fact, many spliced variants of DNMT3B are expressed in a tissue, cell, and/or developmental stage-specific manner [6,9]. For example, DNMT3B1 is highly expressed in human ES cells; in contrast, its expression is decreased in the somatic cells which is accompanied with the predominant expression of DNMT3B3 [33]. Therefore, it is possible that porcine DNMT3B1 might be highly expressed in certain cells but not PAM during the development of pigs.

DNMT3B as one member of DNMTs catalytically regulates de novo DNA methylation. However, not all of DNMT3B isoforms have active catalytic domains. Notable, DNM3B1 and DNMT3B2 have the active catalytic domains; in comparison, DNMT3B3 is considered as an inactive catalytic isoform with some deletions in the catalytic domain. Although the role of DNMT3B3 in catalytic activity is controversial, the related studies have shown that DNMT3B3 could regulate DNA methylation by acting as the accessory protein. In this study, we demonstrated porcine DNTM3B2 regulates methylation of the *TNF-α* gene promoter, which has a role in modulation of expression of TNF-α. Of course, it is not excluded that porcine DNMT3B3 still functions as a positive regulator of DNA methylation. Thus, future studies are needed to answer this question.

## 5. Conclusions

In summary, this study for the first time identified DNMT3B2 as the predominant isoform in PAM. We demonstrated an important role of DNMT3B2-mediated DNA methylation in expression of TNF-α in the LPS-stimulated PAM. In this context, this study could provide evidence that DNMT3B2-mediated DNA methylation is the important mechanism responsible for understanding the inflammatory response in the LPS-stimulated PAM, even possible during some bacterial infections in the lungs of pigs.

## Supplementary Materials

The following are available online at https://www.mdpi.com/2073-4425/11/9/1065/s1, Table S1: Sequence of primers.

## References

[1] Okano M., Bell D.W., Haber D.A., Li E. (1999). DNA methyltransferases Dnmt3a and Dnmt3b are essential for de novo methylation and mammalian development. Cell.

[2] Leonhardt H., Page A.W., Weier H.U., Bestor T.H. (1992). A targeting sequence directs DNA methyltransferase to sites of DNA replication in mammalian nuclei. Cell.

[3] Veland N., Lu Y., Hardikar S., Gaddis S., Zeng Y., Liu B., Estecio M.R., Takata Y., Lin K., Tomida M.W. (2019). DNMT3L facilitates DNA methylation partly by maintaining DNMT3A stability in mouse embryonic stem cells. Nucleic Acids Res..

[4] Manzo M., Wirz J., Ambrosi C., Villaseñor R., Roschitzki B., Baubec T. (2017). Isoform-specific localization of DNMT3A regulates DNA methylation fidelity at bivalent CpG islands. EMBO J..

[5] Ostler K.R., Davis E.M., Payne S.L., Gosalia B.B., Expósito-Céspedes J., Le Beau M.M., Godley L.A. (2007). Cancer cells express aberrant DNMT3B transcripts encoding truncated proteins. Oncogene.

[6] Gopalakrishnan S., Van Emburgh B.O., Shan J., Su Z., Fields C.R., Vieweg J., Hamazaki T., Schwartz P.H., Terada N., Robertson K.D. (2009). A novel DNMT3B splice variant expressed in tumor and pluripotent cells modulates genomic DNA methylation patterns and displays altered DNA binding. Mol. Cancer Res. MCR.

[7] Gopalakrishna-Pillai S., Iverson L.E. (2011). A DNMT3B alternatively spliced exon and encoded peptide are novel biomarkers of human pluripotent stem cells. PLoS ONE.

[8] Weisenberger D.J., Velicescu M., Cheng J.C., Gonzales F.A., Liang G., Jones P.A. (2004). Role of the DNA methyltransferase variant DNMT3b3 in DNA methylation. Mol. Cancer Res. MCR.

[9] Robertson K.D., Uzvolgyi E., Liang G., Talmadge C., Sumegi J., Gonzales F.A., Jones P.A. (1999). The human DNA methyltransferases (DNMTs) 1, 3a and 3b: Coordinate mRNA expression in normal tissues and overexpression in tumors. Nucleic Acids Res..

[10] Qiao S., Feng L., Bao D., Guo J., Wan B., Xiao Z., Yang S., Zhang G. (2011). Porcine reproductive and respiratory syndrome virus and bacterial endotoxin act in synergy to amplify the inflammatory response of infected macrophages. Vet. Microbiol..

[11] Li J., Wang S., Li C., Wang C., Liu Y., Wang G., He X., Hu L., Liu Y., Cui M. (2017). Secondary Haemophilus parasuis infection enhances highly pathogenic porcine reproductive and respiratory syndrome virus (HP-PRRSV) infection-mediated inflammatory responses. Vet. Microbiol..

[12] Yang Q., Pröll M.J., Salilew-Wondim D., Zhang R., Tesfaye D., Fan H., Cinar M.U., Große-Brinkhaus C., Tholen E., Islam M.A. (2016). LPS-induced expression of CD14 in the TRIF pathway is epigenetically regulated by sulforaphane in porcine pulmonary alveolar macrophages. Innate Immun..

[13] Lu Y., Zhang Y., Xiang X., Sharma M., Liu K., Wei J., Shao D., Li B., Tong G., Olszewski M.A. (2020). Notch signaling contributes to the expression of inflammatory cytokines induced by highly pathogenic porcine reproductive and respiratory syndrome virus (HP-PRRSV) infection in porcine alveolar macrophages. Dev. Comp. Immunol..

[14] Brown R.A.M., Epis M.R., Horsham J.L., Kabir T.D., Richardson K.L., Leedman P.J. (2018). Total RNA extraction from tissues for microRNA and target gene expression analysis: Not all kits are created equal. BMC Biotechnol..

[15] Saitou N., Nei M. (1987). The neighbor-joining method: A new method for reconstructing phylogenetic trees. Mol. Biol. Evol..

[16] Felsenstein J. (1985). Confidence limits on phylogenies: An approach using the bootstrap. Evolution.

[17] Lateef S.S., Gupta S., Jayathilaka L.P., Krishnanchettiar S., Huang J.S., Lee B.S. (2007). An improved protocol for coupling synthetic peptides to carrier proteins for antibody production using DMF to solubilize peptides. J. Biomol. Tech. JBT.

[18] Qiu Y., Shen Y., Li X., Ding C., Ma Z. (2008). Molecular cloning and functional characterization of a novel isoform of chicken myeloid differentiation factor 88 (MyD88). Dev. Comp. Immunol..

[19] Xiang X., Zhang Y., Li Q., Wei J., Liu K., Shao D., Li B., Olszewski M.A., Ma Z., Qiu Y. (2020). Expression profile of porcine scavenger receptor A and its role in bacterial phagocytosis by macrophages. Dev. Comp. Immunol..

[20] Neal L.M., Qiu Y., Chung J., Xing E., Cho W., Malachowski A.N., Sandy-Sloat A.R., Osterholzer J.J., Maillard I., Olszewski M.A. (2017). T Cell-Restricted Notch Signaling Contributes to Pulmonary Th1 and Th2 Immunity during Cryptococcus neoformans Infection. J. Immunol..

[21] Li L.C., Dahiya R. (2002). MethPrimer: Designing primers for methylation PCRs. Bioinformatics.

[22] Li L.C. (2007). Designing PCR primer for DNA methylation mapping. Methods Mol. Biol..

[23] von Känel T., Huber A.R. (2013). DNA methylation analysis. Swiss. Med. Wkly..

[24] Liu B., Pilarsky C. (2018). Analysis of DNA hypermethylation in pancreatic cancer using methylation-specific PCR and bisulfite sequencing. Methods Mol. Biol..

[25] Kumaki Y., Oda M., Okano M. (2008). QUMA: Quantification tool for methylation analysis. Nucleic Acids Res..

[26] Kennedy A., Cribbs A.P. (2016). Production and concentration of lentivirus for transduction of primary human T cells. Methods Mol. Biol..

[27] Benskey M.J., Manfredsson F.P. (2016). Lentivirus production and purification. Methods Mol. Biol..

[28] Jain N., Shahal T., Gabrieli T., Gilat N., Torchinsky D., Michaeli Y., Vogel V., Ebenstein Y. (2019). Global modulation in DNA epigenetics during pro-inflammatory macrophage activation. Epigenetics.

[29] Wang H., Feng H., Sun J., Zhou Y., Zhu G., Wu S., Bao W. (2018). Age-associated changes in DNA methylation and expression of the TNF-α gene in pigs. Genes Genet. Syst..

[30] Zhang S., Barros S.P., Moretti A.J., Yu N., Zhou J., Preisser J.S., Niculescu M.D., Offenbacher S. (2013). Epigenetic regulation of TNFA expression in periodontal disease. J. Periodontol..

[31] Kojima A., Kobayashi T., Ito S., Murasawa A., Nakazono K., Yoshie H. (2016). Tumor necrosis factor-alpha gene promoter methylation in Japanese adults with chronic periodontitis and rheumatoid arthritis. J. Periodontal Res..

[32] Okano M., Xie S., Li E. (1998). Cloning and characterization of a family of novel mammalian DNA (cytosine-5) methyltransferases. Nat. Genet..

[33] Liao J., Karnik R., Gu H., Ziller M.J., Clement K., Tsankov A.M., Akopian V., Gifford C.A., Donaghey J., Galonska C. (2015). Targeted disruption of DNMT1, DNMT3A and DNMT3B in human embryonic stem cells. Nat. Genet..

