# Supplementary material for: Identification of DNMT3B2 as the Predominant Isoform of DNMT3B in Porcine Alveolar Macrophages and Its Involvement in LPS-Stimulated TNF-α Expression
Source: Genes (Basel). 2020 Sep 10;11(9):1065. doi: 10.3390/genes11091065 (PMC7564714; doi:10.3390/genes11091065)
Supplement: Supplementary file 1 [file genes-11-01065-s001.pdf]

Supplementary Table 1 Sequence of primers

Primer	Sequence (5′ → 3′)	
DNMT3B 1F	ATGAAGGGAGACACCAGACAACTCAACGG	cloning
DNMT3B 1R	ACCACCATGGCAGGCCACCAGGAGAAAC	cloning
DNMT3B 2F	GAATAGGGGATCTTGTGTGGGGAAA	cloning
DNMT3B 2R	AAAGACACGGGATTTTTCCGACCACA	cloning
DNMT3B 3F	AGATGTGACCAACAATAAGAGCAGT	cloning
DNMT3B 3R	GGGAACTATTCACAGGCAAAGTAGTCCTTCAG GGGGG	cloning
DNMT3B-flag F	TGAACCGTCAGAATTAAGCTTATGAAG GGAGACACCAGACAACTC	homologous recombination
DNMT3B-flag R	TTTGTAGTCAGCCCGGGATCCTTCA CAGGCAAAGTAGTCCTTCAGG	homologous recombination
TNF-α F	AGAAGGATGATCGACTCA	qPCR
TNF-α R	AAATAGACCTGCCCAGAT	qPCR
GAPDH F	TCTGGCAAAGTGGACATT	qPCR
GAPDH R	GGTGGAATCATACTGGAACA	qPCR
DNMT3B F	CAGACAATAACCACCAAGT	qPCR
DNMT3B R	ACATCTTCTTTGCCATTCA	qPCR
DNMT1 F	CATAAGTAAGATAGTGGTTGAGTT	qPCR
DNMT1 R	CACAGAAGGAGGAACAGT	qPCR
DNMT3A F	GTAAGTGATGCTCGTGACATAG	qPCR
DNMT3A R	TGATGAAGGCGGAACTGA	qPCR
Exon10 F	CCCCCCCGAAAAAAGTGGTTAATAA	PCR
Exon10 R	GAATGTGCAGAGAAAGTAGGGCCAA	PCR
Exon10-RT-F	GGTCGAGGGGCTCAAACCCAACAACAAGC	RT-PCR
Exon10-RT-R	GCTTGGGTGGGGGGCAGTAGTCAGAGG	RT-PCR
Exon21/22-F	AGAGCCCGATATTTCTGG	RT-PCR
Exon21/22-R	TGGAGACATCTGTGTAGTG	RT-PCR
TNF-α-bsp-F	ATGTTTGGGTGTTTTTAATTTTTTAAAT	BSP
TNF-α-bsp-R	CTAACTAATCCCCTAATATCCTCACTCTA	BSP
